# Coexistent sickle-cell anemia and autoimmune disease in eight children: pitfalls and challenges

**DOI:** 10.1186/s12969-017-0221-x

**Published:** 2018-01-17

**Authors:** Valerie Li-Thiao-Te, Florence Uettwiller, Pierre Quartier, Florence Lacaille, Brigitte Bader-Meunier, Valentine Brousse, Mariane de Montalembert

**Affiliations:** 1 Onco-Hématologie Pédiatrique, Centre Hospitalier Universitaire Amiens, Amiens, France; 2 Unité d’Immunologie-Hématologie et Rhumatologie Pédiatrique, Centre de référence pour la Rhumatologie et les maladies Auto-immunes Systémiques de l’Enfant, Hôpital Necker-Enfants Malades, Assistance Publique Hôpitaux de Paris, Paris, France; 3 Université Paris-Descartes, Paris, France; 4 IMAGINE Institute, Paris, France; 5 Hépatologie-Gastroentérologie-Nutrition Pédiatrique, Hôpital Necker-Enfants Malades, Assistance Publique Hôpitaux de Paris, Paris, France; 6 Pédiatrie Générale, Centre de Référence des Hémoglobinopathies, Hôpital Necker-Enfants Malades, Assistance Publique Hôpitaux de Paris Labex-GR-Ex, Paris, France

**Keywords:** Sickle cell disease, Autoimmune disease, Juvenile idiopathic arthritis, Systemic lupus erythematosus, Children, Biological therapy

## Abstract

**Background:**

Patients with sickle cell disease (SCD) present a defective activation of the alternate complement pathway that increases the risk of infection and is thought to predispose to autoimmune disease (AID). However, coexisting AID and SCD is rarely reported, suggesting possible underdiagnosis due to an overlapping of the symptoms.

**Study design:**

Among 603 patients with SCD followed between 1999 and June 2016, we retrospectively searched for patients with coexisting SCD and AID.

**Results:**

We identified 8 patients aged from 7 to 17 years diagnosed with AID; juvenile idiopathic arthritis (*n* = 3), systemic lupus erythematosus (*n* = 2), Sjögren’s syndrome (*n* = 1) and autoimmune hepatitis (n = 2). The diagnosis of AID was often delayed due to similarities of the symptoms with those of SCD. Patients treated with steroids experienced multiple vaso-occlusive crises and received prophylactic chronic blood transfusions when it was possible. Tolerance to other immunosuppressive and biological treatments, such as anti-TNF agents, was good. A remission of AID was achieved in 4 patients, without worsening the course of the SCD. One patient underwent a geno-identical hematopoietic stem cell transplantation that cured both diseases. Another one underwent a successful liver transplantation.

**Conclusion:**

Coexistence of AID and SCD generates diagnostic and therapeutic challenges. Early diagnosis of AID is important to define the best treatment, which may include targeted biological therapy.

## Background

Sickle cell disease (SCD) is a common inherited condition in African, Caribbean, and Mediterranean countries. Production of abnormal hemoglobin S is responsible for the sickling of red blood cells in deoxygenated conditions. SCD manifests itself as chronic intravascular hemolysis, vaso-occlusion and painful crises. Over time, multiple organ damage can develop. Patients with SCD display a defective activation of the alternate complement pathway, leading to an increased risk of infection with encapsulated bacteria (pneumococci, *Haemophilus influenzae*) [1–3]. It was suggested that complement abnormalities could predispose to autoimmune disease (AID) [4]. However, the frequency of coexisting SCD and AID has not been evaluated, and most data come from case reports [5–14]. Manifestations of AID, such as fever, polyarthritis, and multiorgan involvement, can also be caused by SCD, leading to a delay in diagnosing the AID [11]. Furthermore, antiinflammatory drugs such as steroids and immunosuppressive therapy used to treat AID can induce severe SCD complications.

## Study design

Between 1999 and June 2016, 603 patients with SCD aged under 18 years were seen at the hemoglobinopathy reference center of the Necker Hospital, Paris, France. Among them, we searched for patients with coexisting SCD and AID.

We retrospectively collected data from the medical records of SCD patients followed in our institution, the largest Pediatric University-Hospital in France, which has a national reference center for SCD and a national reference center for pediatric rheumatology diseases. Started in 2007, data from the patients referred to the pediatric rheumatology team were registered in the CEMARA database, a nationwide web-based information for rare diseases [15] approved by the French patients Data Protection Authority (*Commission Nationale de l’Informatique et Libertés*, CNIL), after patients’/parents’ information and verification of their non-opposition. In accordance with the French legislation, no ethics committee agreement was requested for such a retrospective survey.

## Results

Among the 603 children with SCD followed between 1999 and June 2016, we identified 8 patients aged from 7 to 17 years, who developed an AID. All patients were Afro-Caribbean homozygous SCD (Hb SS) children either diagnosed by neonatal screening (patient 1, 6, 7 and #8) or during infancy (patient 2, 3, 4 and #5). The diagnoses were juvenile idiopathic arthritis (JIA, *n* = 3), systemic lupus erythematosus (SLE, *n* = 2), Sjögren’s syndrome (*n* = 1) and autoimmune hepatitis (n = 2). These 8 patients received joint follow-up by hematologists, rheumatologists and an hepatologist. Table 1 reports the medical history before the diagnosis of the AID and the clinical and laboratory findings that led to the diagnosis of AID.

**Table 1:** Medical history, clinical and biological findings at presentation in our patients

Patient	1	2	3	4	5	6	7	8
Sex	M	M	F	M	M	F	F	F
Medical history related to SCD	ACS (3 episodes) Recurrent VOC	Recurrent polyarthralgia^a^	Recurrent VOC	Recurrent VOC Osteomyelitis right femur Chronic hepatitis B	Recurrent VOC Acute cholecystitis, *Salmonella* septicemia Bilateral avascular necrosis of femoral heads	Recurrent VOC	Recurrent VOC ACS (3 episodes)	Recurrent VOC ACS Acute splenic sequestration Recurrent cholangitis
Type of AID	SLE	JIA	JIA	JIA	AIH	Sjogrën’syndrome	SLE	AIH
Age at onset of AID (years)	13	4	7	4	17	11	15	3
Age at diagnosis of AID (years)	13	13	7	6	17	13 (11 for initial diagnosis of JIA)	15	3
Symptoms	Asthenia, denutrition polyarthritis fingers and toes hepatomegaly pericarditis	Polyarthritis left knee and right ankle severe arthropathy at 10 y uveitis	Polyarthritis (temporo-mandibular arthritis and sacroiliitis)	Polyarthralgia bilateral knee arthritis Asthenia, weight loss	Polyarthralgia	Polyarthralgia (spine); knee arthritis; Secondary recurrent parotiditis; xerostomia at 13 y	Polyarthritis; pleuritis, pericarditis, aphthous stomatitis, alopecia; pulmonary hypertension; behavior disorder	Hepatomegaly; jaundice
ESR (mm/h)	74	95	34	86	67	14	84	88
IgG (g/l)	38	26	–	–	35	23	30	26
ANA pattern	1/1280 homogeneous	1/160 speckled	Negative	1/80 homogeneous	1/80	1/1280	1/80 speckled	1/160
Others immunological features	Anti-DNA antibodies + (43 UI/ml) Coombs +	RF negative	RF positive		Anti-U1RNP + Farr test 29%	Anti-SSA, SSB + Anti-U1RNP + Antiβ2GP1 + Antiphospholipid antibodies +	Anti-DNA antibodies + (44 U/l) pANCA Coombs + Antiplatelets antibodies + AntiB2GP1 + anticardiolipin + Neutropenia	c-ANCA + Smooth muscle antibodies + (1/50)

*VOC* vaso-occlusive crises, *ACS* acute chest syndrome, *AID* autoimmune disease, *SLE* systemic lupus erythematosus, *JIA* juvenile idiopathic arthritis, *AIH* autoimmune hepatitis, *M* male, *F* female, *ESR* erythrocyte sedimentation rate, *ANA* antinuclear antibodies, *RF* rheumatoid factor

^a^first ascribed to SCD but probably related to JIA

In one patient, SLE was diagnosed within a few weeks of the onset of the symptoms because of fingers swelling, not a common site of vaso-occlusion in adolescents with SCD. In another patient, alopecia and aphthous stomatitis helped diagnose the SLE several months after the first neurologic symptoms and polyarthritis had appeared. In some patients, the diagnosis was delayed for several years (Table 1). Before the diagnosis of JIA, patients #2 and #4 had had a long history of joint pain and ankle and knee swelling, first ascribed to SCD and treated as SCD complications (analgesics, hyperhydratation, hydroxyurea for patient #2 and exchange transfusion for patient #4). Patients 5 and #8 displayed biological signs of liver dysfunction (elevation of AST and ALT, cholestasis). Liver biopsy showed lymphoplasmocytic infiltrates and mild fibrosis compatible with autoimmune hepatitis. For patient #7 diagnosed with SLE, cerebral MRI was performed in response to the worsening of neurologic symptoms and showed periventricular hyperintensity and cortico-subcortical atrophy compatible with lupus lesions. The tolerance to AID treatment was good. Among the 4 patients treated with steroids, 3 had a concomitant exchange transfusion program in order to prevent vaso-occlusive crises and 1 received hydroxyurea. Red blood cell alloimmunisation in 1 patient required a switch from transfusion to hydroxyurea. Complete remission of the AID was obtained for 4 patients with a classical immunosuppressive treatment (*n* = 1), biological treatment (*n* = 2) or genoidentical hematopoietic stem cell transplantation (n = 1), although 1 patient (patient #4) had arthritis sequelae. Despite being associated with AID, the SCD course was stable for 5 patients. The patient who underwent stem cell transplantation was cured of both AID and SCD. Patient #4 who did not receive hydroxyurea or transfusion experienced several vaso-occlusive crises during the follow up. Patient #8 who underwent liver transplantation due to chronic infectious cholangitis, presented multiple post-transplant complications (rejection, biliary stenosis, high blood pressure, posterior reversible encephalopathy), but had a normal liver function and a much improved quality of life at the last follow up. Table 2 reports the treatments and last follow-up data.

**Table 2:** Treatment and last follow-up in the 8 patients

Patient	1	2	3	4	5	6	7	8
Treatment related to SCD before the diagnosis of AID	None	Hydroxyurea started at 13 y	Hydroxyurea started at 7 y	None	None	Hydroxyurea started at 9 y	Hydroxyurea started at 14 y	Chronic exchange transfusion programme started at 3 y splenectomy
AID	SLE	JIA	JIA	JIA	AIH	Sjogrën’s syndrome	SLE	AIH
Treatment of AID First line	NSAIDs Hydroxychloroquine	NSAIDs	NSAIDs for 3 years	Etanercept (No methotrexate because of chronic hepatitis B)	Steroids Chronic blood transfusions then Hydroxyurea^a^	NSAIDs (from age 11 to 13 y)	Steroids Chronic exchange transfusions^a^ Hydroxychloroquine Aspirin	Steroids 6-mercaptopurine Chronic exchange transfusions^a^
2nd line	Genoidentical hematopoietic stem cell transplantation at 18 y	Etanercept	Steroids Intra-articular steroids		Azathioprine NSAIDs	Methotrexate Hydroxychloroquine Aspirin	Mycophenolate mofetil Steroids	Azathioprine Steroids Chronic exchange transfusions^a^
3rd line and more			Etanercept for 5 years; (Failure of abatacept, infliximab, adalimumab) Current treatment: Tolicizumab + methotrexate			Mycophenolate mofetil		Liver transplantation at 10 y Ciclosporin and steroids
Last follow-up	CR	PR	Persistant activity	CR	CR	CR	PR	Normal liver function Controlled blood pressure

*AID* autoimmune disease, *SLE* systemic lupus erythematosus, *JIA* juvenile idiopathic arthritis, *AIH* autoimmune hepatitis, *NSAIDs* non steroidal antiinflammatory drugs, *CR* complete remission, *PR* partial remission

Patient 2: relapse after 3 years, then lost in transition to adult care, patient 7: persistent periventricular hyperintensity on cerebral MRI

^a^in order to prevent occurrence of vasoocclusive crises

## Discussion

We report 8 cases of coexisting SCD and AID including polyarticular JIA and juvenile SLE in a cohort of 603 patients with SCD. The incidence of AID in France in an ethnic matched population is not known, as it is not allowed to compare diseases according to patients’ ethnicity. These cases highlight the diagnostic difficulties raised by the symptom overlapping between AID and SCD (e.g., polyarthritis, anemia, and fever) and the therapeutic challenges. Nevertheless, the use of immunosuppressive drugs (except steroids), including new drugs such as TNFα antagonist was well tolerated and provided durable AID remissions, without worsening the course of the SCD when associated to specific SCD treatments.

AID such as SLE [16] and polyarticular JIA [17, 18] are more common in Blacks than in other ethnic groups. In addition to genetics, patients with SCD present functional asplenia and defective activation of the alternate complement pathway [1–4]. Although published data focused on functional complement abnormalities, it would be interesting to assess the impact of functional asplenia itself in the occurrence of AID [19]. However, the coexistence of AID and SCD seems to be rare, potentially underdiagnosed, and its prevalence is unknown. In a study of 30 patients with SCD and connective tissue disease, 15 patients (50%) had rheumatoid arthritis, 13 (44%) had SLE, 1 (3%) had Sjögren’s syndrome, and 1 (3%) had sclerodermia [20]. Most data on coexistent AID and SCD come from anecdotal case-reports [5–14] describing patients SLE [2, 8–11], or far less often, JIA [5–7] or autoimmune hepatitis [12–14].

Our patients reflect the diagnostic challenges of JIA in children with SCD. We report diagnostic delays from 1 to 8 years, as could be found in previous reports [5–7]. Features arguing against SCD as the cause included bilateral symmetric pain limited to the joints, failure of hydroxyurea, persistent high erythrocyte sedimentation rate. In most previously reported cases, the recognition of SLE was delayed because of similar clinical features, regardless of involved organs [8–10]. The liver involvement is often misdiagnosed because jaundice is ascribed to hemolysis and not cholestasis.

An autoantibody production is traditionally a manifestation of autoimmunity. Nearly all our patients had detectable antinuclear antibodies. Whether early autoantibody detection might have helped to diagnose AID is discussed. The prevalence of antinuclear antibodies varies from 12 to 30% in healthy individuals overall [21, 22] and from 7 to 39% in Africans [23, 24]. Moreover, high autoantibody titers have been reported in SCD in the absence of autoimmunity [25, 26]. Consequently, routine screening for autoantibodies in the absence of suggestive symptoms is not recommended, because it may be confusing and lead to inadequate diagnosis and therapy. The mechanisms underlying the production of autoantibodies in SCD are unknown but may involve impaired spleen function. Significant autoantibody titers have been found after splenectomy, in the absence of AID [27]. Other hypotheses include immune system overstimulation by multiple transfusions [28] and chronic inflammation [29]. This has not been confirmed in clinical studies [25, 26].

The treatment of coexisting AID and SCD is difficult [30]. Our patients experienced recurrent vaso-occlusive crises while on steroids [7, 10, 13, 30]. Concomitant red blood cell transfusion or exchange transfusion program may help prevent vaso-occlusive crises but increase the risk of anti-erythrocyte alloimmunization. Half of our patients received hydroxyurea in the first line or after chronic blood transfusion and experienced fewer vaso- occlusive crises. Hydroxyurea seems to be well tolerated by patients taking antiinflammatory drugs [8–10]. TNFα antagonist therapy (etanercept) was promptly effective in our patients with JIA, as reported in patients with polyarticular onsets of JIA and no SCD [31, 32]. No severe etanercept-related complications were recorded. The few available data suggest good effectiveness and tolerance in patients with SCD [5, 20]. The use of others biological therapies as anti-interleukine 1 and 6 has not been reported yet in patients with SCD. Azathioprine was frequently used either for patients with SLE or autoimmune hepatitis and was well tolerated [10, 12–14]. For hydroxychloroquine, ocular toxicity may worsen retinopathy due to SCD and has to be regularly controlled. Finally, immunosuppressive and antiinflammatory drugs can induce renal and hepatic toxicity that may be worsened by SCD-related complications. They may further increase the risk of infection [20], despite the lack of published data concerning infections in patients with both AID and SCD. The initiation of immunosuppressive and/or antiinflammatory therapy should be discussed in reference centers and require close monitoring. Biological treatments, including anti-TNF targeted treatments, might be beneficial to SCD-associated AID / inflammation and in some cases to SCD itself and deserve further evaluation [33]. Hematopoietic stem cell transplantation seems to be a good therapeutic option. Liver transplantation can be discussed in very selected cases.

## Conclusion

SCD and AID share many clinical and biological features, and diagnosing AID in patients with SCD can be challenging. Diagnostic delay increases the morbidity and mortality related to both the AID and SCD. Whilst the use of steroids to treat AIDs induced potential severe SCD-related complications, which justified associated chronic red blood cell transfusions, other treatments such as anti-TNF agents were well tolerated. Further studies are needed to determine the prevalence of AID in patients with SCD, to look for pathophysiological links, to earlier diagnose AID, and to define the best therapeutic options.

## Abbreviations

ACS: Acute chest syndrome
AID: Autoimmune disease
AIH: Autoimmune hepatitis
CNIL: Commission Nationale Informatique et Libertés
JIA: Juvenile idiopathic arthritis
SCD: sickle cell disease
SLE: Systemic lupus erythematosus
VOC: Vaso-occlusive crises
